# Ecological and Genetic Landscapes of Global H12 Avian Influenza Viruses and Biological Characteristics of an H12N5 Virus Isolated from Wild Ducks in Eastern China

**DOI:** 10.1155/2024/9140418

**Published:** 2024-02-22

**Authors:** Mengjing Wang, Jing Guo, Hong Zhang, Xiaohong Sun, Jinyan Shen, Mengdi Guan, Lili Liu, Wenqiang Liu, Zhijun Yu, Anran Ren, Yubao Li, Xuyong Li

**Affiliations:** ^1^College of Agronomy, Liaocheng University, Liaocheng, China; ^2^Poultry Institute, Shandong Academy of Agricultural Sciences, Shandong, China

## Abstract

Wild migratory birds are considered the central reservoirs of avian influenza viruses. H12 viruses are one of the 16 hemagglutinin (HA) subtypes of avian influenza viruses and are rarely reported because they are infrequently detected in birds. Consequently, the ecological and genetic profiles of H12 viruses and their adaptation in domestic birds and mammals remain unclear. Here, we found that H12N5 viruses were predominant in the nine identified H12NX subtypes, with the HA (H12) and neuraminidase (NA) (N5) genes showing combination bias in the categorized analysis of subtype combinations (H12 and N1–N9; H1–H12, H14, H15, and N5). These identified H12N5 viruses were primarily detected in birds of *Anatidae* and *Scolopacidae* in North America, excluding their possible characterization as chicken or mammalian viruses. The H12N5 viruses were divided into the North American lineage and Eurasian lineage according to their genetic differences, including the HA and NA surface genes and internal genes, although reassortment was observed between the two lineages. We isolated an Eurasian-lineage H12N5 virus from wild ducks in Eastern China, which was one of the 12 identified H12 viruses in China. Infectivity studies indicated that the H12N5 virus is poorly adapted to domestic ducks and chickens, although viral shedding could be detected in both inoculated and contact birds. Additionally, the naturally isolated H12N5 virus did not achieve good replication in mice. These results indicate that the rare subtype of H12 viruses was mainly pooled in wild migratory birds and has an established phylogeography, with low risks of spillover into domestic birds and mammals.

## 1. Introduction

Influenza A viruses are members of the Orthomyxoviridae family that contain eight negative-sense RNA segments. Influenza A viruses have a wide host range, including wild birds, poultry, mammals, and humans. To date, 16 hemagglutinin (HA) and nine neuraminidase (NA) influenza A viruses have been identified in birds. Avian influenza viruses (AIVs) are influenza A viruses and have been closely associated with influenza pandemics over the past century [1–4]. The emergence or reemergence of AIVs in the past 2 decades, including the highly pathogenic H5Nx and H7N9 viruses and the lowly pathogenic H9N2, H3N8, H10N3, H10N8, and H7N4 viruses, posed a continuing threat to animal and human health [5–19].

Wild waterfowl and shorebirds are recognized as the major reservoirs of the rare subtypes (H8, H11, and H12–H16) of AIVs and play a key role in the maintenance of the gene pool of AIVs [20]. H12 AIV was first detected in a gadwall in America in 1975 (A/gadwall/Wisconsin/13/1975 (H12N5)) and is rarely reported because of its infrequent detection in birds. H12 viruses are primarily found in migratory waterfowl and shorebirds and have not been found in chickens, turkeys, or mammals. Several studies have reported the isolation of H12 viruses, including the H12N2 and H12N5 viruses, from migratory birds and performed genetic and phylogenetic analyses [14, 21–23]. The H12N2 reassortant containing the gene segments of North American lineage and Eurasian lineage was identified from common teal in the Russian Far East in 2018 [21]. Previous studies also reported that H12 viruses showed high attachment to *α*2–3-linked SA structures, which represented the primary binding to avian-type receptors [23, 24]. Due to the infrequent detection of H12 viruses, the characteristics of their subtypes, including ecology, phylogeography, and replication ability in birds and mammals, are largely unknown. As one of the nine neuraminidase subtypes, N5 can combine with different HA subtypes and has been reported as the donor of emerged highly pathogenic H5N5 avian viruses, but the detection rate of N5 is relatively lower compared with those common NA subtypes [25–29].

Annual active AIV surveillance has been conducted in the Yellow River Delta (YRD) wetland since 2017. The YRD wetland located in Eastern China is an important habitat for migratory birds on the East Asian-Australasian (EAA) flyway. We have identified 28 subtypes of AIVs in wild birds in YRD wetland from 2017 to 2022, as shown in our previous studies [8, 9, 30–35]. In this study, we fully uncovered the ecology and evolutionary dynamics of the globally identified H12 viruses. We isolated an H12N5 virus from a migratory duck and evaluated its infection and replication in domestic birds and mice. These results will contribute to our comprehensive understanding of the evolution and potential infection risks of rare H12 viruses.

## 2. Materials and Methods

### 2.1. Sampling and Virus Isolation

Field surveys and sample collection were conducted during the migration season of the birds in the YRD wetland, which is located in the Shandong Province of Eastern China. The wild bird species were first confirmed by drone photography or camera photography. Fresh fecal droppings were then placed into 2 ml of minimal essential medium supplemented with penicillin and streptomycin. The fecal samples were identified by PCR with specific M and HA primers for AIVs (M-F: AGCRAAAGCAGGTAGATRTTKAAA; M-R: AGTAGAAACAAGGTAGTTTTTTA; HA-F: AGCAAAAGCAGGGG; HA-R: AGTAGAAACAAGGGTGTTTT). The suspected positive samples were injected into 10-day-old embryonated chicken eggs for virus isolation in an ABSL-2 laboratory at Liaocheng University. An HA assay was performed with 0.75% chicken red blood cells to confirm the presence of influenza viruses in the allantoic fluid of the eggs. The viruses were stored in a −80°C freezer. The HA and NA subtypes of all the viruses were identified by viral genome sequencing. The virus A/wild duck/Shandong/W6808/2019 (H12N5) used in this study was isolated from fecal droppings of wild ducks in Eastern China.

### 2.2. Data Acquisition

To fully uncover the ecology and epidemiology of the global H12 viruses, the HA sequences of H12 viruses were downloaded from the Global Initiative on Sharing All Influenza Data (GISAID) and GenBank (National Center for Biotechnology Information, NCBI) public databases. The available virus information, including the subtype combinations, hosts, and temporal and spatial distributions, of these identified H12NX viruses was classified.

### 2.3. Phylogenetic Analysis

A phylogenetic tree of HA and NA genes of the H12N5 viruses and their internal genes (PB2, PB1, PA, NP, M, and NS) was constructed using BEAST (v1.10.4), as described previously [36]. Briefly, all the sequences downloaded from the databases and the sequences of the virus detected in this study were first aligned using MAFFT (a multiple sequence alignment program) [37], and then the aligned sequences were screened in MEGA 11. For all eight datasets, sequences without the full alignment length were excluded. By utilizing the model of nucleotide substitution selected in ModelFinder of IQ-TREE and setting uncorrelated relaxed lognormal clocks in the BEAUti program in the BEAST package, phylogenetic trees were generated with the Bayesian Markov chain Monte Carlo (MCMC) method. The best-fitting nucleotide model GTR + F + I+ G4 for HA data was selected, representing the distribution rate change across sites, and the MCMC chain was run 2 × 10^7^ times and sampled every 10,000 steps to generate a beast file. The best-fitting nucleotide model GTR + F + G4 for NA data was selected, and the MCMC chain was run 3.6 × 10^7^ times and sampled every 10,000 steps to generate a beast file. The best-fitting nucleotide model GTR + F + G4 was used for the PB1, PA, NP, M, and NS genes, while the best-fitting nucleotide model for PB2 was GTR + F + I + G4. The MCMC chains for PB1, PA, M, and NP were run 5 × 10^7^ times, that for PB2 run 1.35 × 10^8^ times, and that for NS run 1.8 × 10^8^ times. Tracer (v1.7.1) was used to observe whether the parameters converged (effective sample size values ≥200). A target tree was obtained by selecting the tree with the largest posterior probability with a 10% burn-in by using the Tree Annotator program in the BEAST package. The ML trees were visualized and beautified by Figtree (v1.4.4).

### 2.4. Facility and Animal Infection Studies

The infection studies of H12N5 virus in mice, chickens, and ducks were approved by the Committee on the Ethics of Animal Experiments of Liaocheng University and were carried out in strict accordance with the recommendations in the Guide for the Care and Use of Laboratory Animals of the Ministry of Science and Technology of the People's Republic of China (M-INF-2023-03 (mice); CK-INF-2023-05 (chickens); DK-INF-2023-01 (ducks)). The infection studies were conducted in an animal biosafety level 2 (ABSL-2) laboratory at Liaocheng University. Six-week-old specific pathogen-free (SPF) female BALB/c mice were purchased from Jinan Pengyue Experimental Animal Breeding Co., Ltd. (Shandong, China). Six-week-old SPF chickens and four-week-old SPF ducks were purchased from Shandong Healthtech Laboratory Animal Breeding Co., Ltd. (Shandong, China).

### 2.5. Ducks

Three SPF ducks were inoculated with 10^6^EID_50_ of the tested H12N5 virus in a volume of 200 *µ*l. The birds were euthanized on day 3 p.i., and tissue samples (trachea, lung, pancreas, liver, spleen, kidney, intestine, rectum, and bursa of Fabricius) were collected for viral titration in eggs. To evaluate the transmission of the H12N5 virus in ducks, three SPF ducks were inoculated with 10^6^EID_50_ of the virus in a volume of 200 *µ*l, and three naive ducks were placed into the same isolator as the contact group at 24 hpi. Oropharyngeal and cloacal swabs were collected on days 1, 3, 5, 7, 9, and 11 p.i. for virus titration in eggs. The sera of the inoculated and contact ducks were collected on days 10, 15, and 21, respectively, and antibody titers against the H12N5 virus used in this study were determined by HI test. Briefly, the sera samples were diluted by serial twofold dilution and then reacted with the 4-unit viral antigen (H12N5 virus), 1% RBC (red blood cells) were added to determine the HI titers.

### 2.6. Chickens

Nine SPF chickens were used to test the replication and transmission of the H12N5 virus in chickens. The methods were consistent with those used for the duck study, as described above.

### 2.7. Mice

Ten 6-week-old female SPF mice were inoculated intranasally with 10^6^EID_50_ of the virus in a volume of 50 *µ*l. Three mice were euthanized on day 3 p.i., and brain, spleen, kidney, lung, and nasal turbinate samples were collected for further viral titration in eggs. Lung samples from two additional mice were collected and fixed in 10% formalin and then stained with hematoxylin and eosin (H&E) for histological analysis. The remaining five mice were continuously monitored for body weight changes and survival for 14 days. Five mice inoculated with PBS were used as the control group to observe body weight changes.

## 3. Results

### 3.1. Prevalence and Ecology of H12 AIVs in Birds

**Figure 1:**
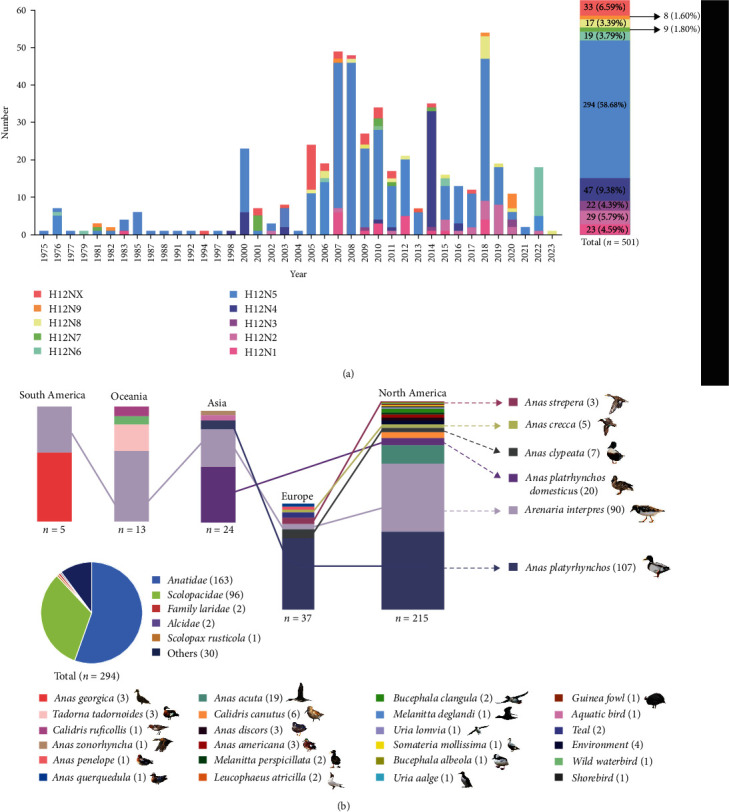
Ecology and temporal and spatial distributions of global H12 viruses. (a) Numbers of HA sequences of each subtype combination of H12 and NA (N1–N9, or unidentified NA subtype) in influenza virus sequence databases (GenBank and GISAID). (b) Geographical distribution of global H12N5 viruses and their reservoirs. The isolation region and the host information of these identified H12N5 viruses were collected from the deposited sequences in Genbank or GISAID. “X” of H12NX indicates the unidentified subtype of NA genes of the viruses in the databases. All the public data in GenBank and GISAID used in this study were up to date as of May 31, 2023.

Knowledge of the prevalence and ecology of H12 viruses is incomplete. Summarization and categorization of virus information for the available strains will contribute to our understanding of the ecology and epidemiology of the viruses. Since 1975, a total of 501 H12 viruses, including nine HA and NA (N1–N9) subtype combinations, have been found worldwide (HA and NA sequences are available from the GenBank and GISAID databases). Importantly, more than 58% (*n* = 294) of the identified H12 viruses (*n* = 501) were subtypes of H12N5 (Figure 1(a)). We then categorized the information of the known H12N5 viruses (*n* = 294), including their hosts and locations. Globally, H12N5 viruses have been detected in North America (*n* = 215), Europe (*n* = 37), Asia (*n* = 24), Oceania (*n* = 13), and South America (*n* = 5). The identified H12N5 viruses were found in 30 species of birds, predominantly birds of *Anatidae* (*n* = 163) and *Scolopacidae* (*n* = 96) (Figure 1(b)). Of note, North American viruses have been detected in at least 21 species of birds, mainly mallards (*Anas platyrhynchos*) and ruddy turnstones (*Arenaria interpres*). The European H12N5 viruses were mainly detected in mallards, while the Asian viruses were primarily found in domestic ducks (*A. platyrhynchos domesticus*) and ruddy turnstones (Figure 1(b)). The H12 virus was first detected in China in 2011, and a total of 12 viruses, including H12N2, H12N5, H12N7, and H12N8, have been reported in wild ducks and wild geese to date. No poultry or mammalian H12 viruses have been reported in China (Table 1). Taken together, these findings indicate that wild ducks and ruddy turnstones are the main natural reservoirs of H12N5 viruses. These data will contribute to our targeted surveillance of waterfowl or shorebirds.

### 3.2. Combination Bias of HA and NA (N5) in Animals

**Figure 2:**
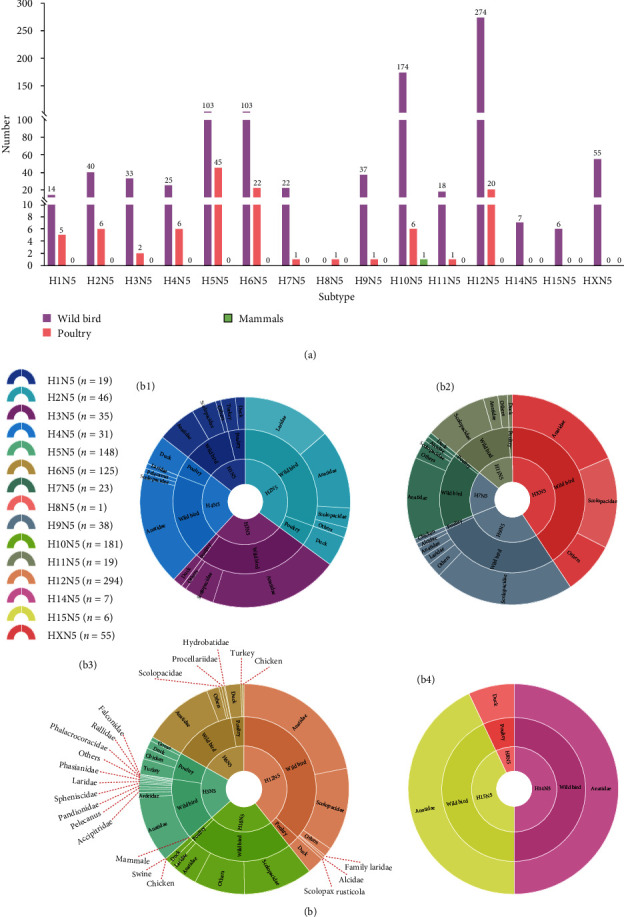
Prevalence of HXN5 viruses in animals. (a) Summarized data of AIVs of subtypes HA (H1–H16) and N5 in wild birds, poultry, and mammals. (b) Specified hosts of HXN5 (H1–H16) viruses. (b1) H1N5, H2N5, H3N5, and H4N5. (b2) H7N5, H9N5, H11N5, and HXN5. (b3) H5N5, H6N5, H10N5, and H12N5. (b4) H8N5, H14N5, and H15N5. The number of each subtype and their host information were from Genbank or GISAID. H13N5 or H16N5 virus has not been reported and their sequences were not available from Genbank or GISAID. All the public data in GenBank and GISAID used in this study were up to date as of May 31, 2023.

The HA and NA of each subtype of the influenza viruses have different combination biases. Here, we summarized the avian influenza strains containing the N5 genes and clarified their distribution in wild birds, poultry, and mammals. All the available viruses of the N5 subtype were classified into 14 HA (H1–H12, H14, and H15) combinations. The subtypes of H13N5 and H16N5 in theory have not been reported until now. Most of the 14 HA and NA subtype combinations, except for H5N5, H6N5, H10N5, and H12N5 viruses, are rarely found in birds and mammals (Figure 2(a)). Importantly, most of the identified viruses of the HXN5 subtype were detected in wild birds, including wild waterfowl (*Anatidae*) and shorebirds (*Scolopacidae* and *Laridae*). In summary, the H3N5, H4N5, H5N5, H6N5, and H7N5 viruses and the rare subtypes of H8N5, H14N5, and H15N5 viruses were primarily discovered in birds of *Anatidae*, while the H9N5, H10N5, and H11N5 viruses dominantly circulated in shorebirds of *Scolopacidae*. The H12N5 viruses and the rare subtypes of H1N5 and H2N5 viruses were primarily found in both waterfowl (*Anatidae*) and shorebirds (*Scolopacidae* or *Laridae*) (Figure 2(b)). These results suggested that H12N5, H10N5, H5N5, and H6N5 predominantly found in waterfowl and shorebirds are the common subtype combinations of HA and NA (N5).

### 3.3. Evolution and Genetic Diversity of H12N5 Viruses

**Figure 3:**
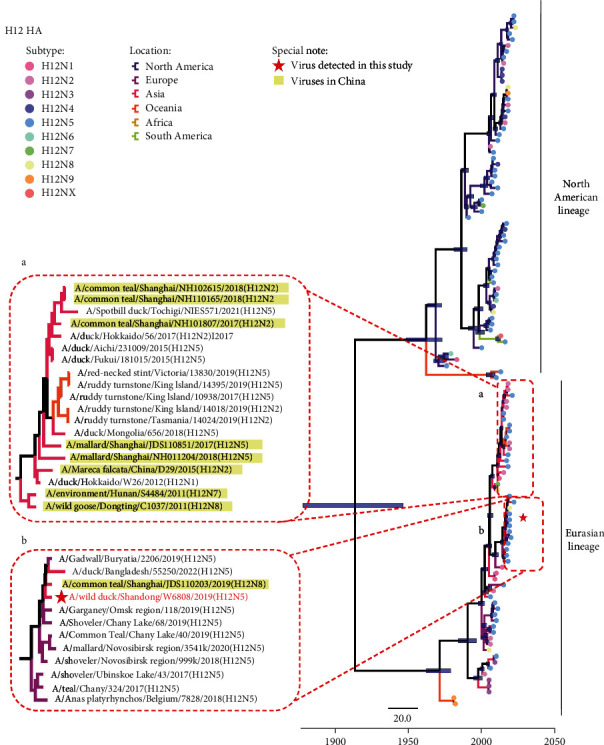
Phylogeography of HA of the H12 viruses. Bayesian time-resolved phylogenetic tree of HA genes of the H12 viruses from 1975 to 2022 (*n* = 117). The colored branches in the phylogenetic tree indicate the isolated locations of the viruses, and the tip points indicate the subtypes of the viruses. The H12 viruses detected in China and the virus used in this study are marked. “X” of H12NX indicates the unidentified subtype of NA genes of the viruses in the databases.

**Figure 4:**
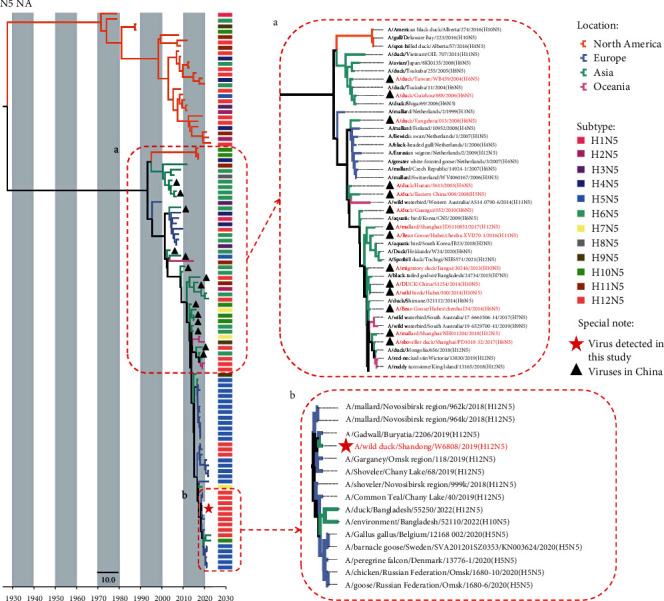
Bayesian time-resolved phylogenetic tree of NA (N5) genes. Maximum clade credibility (MCC) trees were constructed for the NA genes (*n* = 104) of HXN5 viruses (H1N5 (*n* = 2), H2N5 (*n* = 3), H3N5 (*n* = 3), H4N5 (*n* = 6), H5N5 (*n* = 24), H6N5 (*n* = 18), H7N5 (*n* = 3), H8N5 (*n* = 1), H9N5 (*n* = 3), H10N5 (*n* = 7), H11N5 (*n* = 6), and H12N5 (*n* = 28)). The locations, subtypes of the viruses, viruses detected in China, and viruses used in this study are specifically noted.

Bayesian time-resolved phylogenetic trees were constructed to reveal the evolutionary trend of the HA and NA surface genes of the H12N5 viruses. The HA genes of all the available H12 viruses evolved into two lineages that are closely related to the spatial distribution of the viruses. The viruses of the North American lineage were mostly detected in North America, plus a few South American viruses, while the H12 viruses collected from birds in Europe, Asia, Oceania, and Africa clustered into the Eurasian lineage. The H12 viruses detected in China share a high genetic identity with the Asian and European viruses (Figure 3). Notably, the A/wild duck/Shandong/W6808/2019 (SD/W6808) H12N5 virus isolated in Shandong Province shared high genetic similarity with the H12N2 virus detected in a common teal in Shanghai, which is located on the same migratory flyway of Eastern China (Figure 3) [22]. The phylogenetic analysis of NA genes of subtype N5 indicated that the North American HXN5 viruses were genetically different from the viruses in Eurasia and Oceania (Figure 4). However, the H4N5 and H10N5 viruses containing N5 genes of Eurasian viruses were once detected in North America in 2016, although these viruses may be transient. The NA genes of the HXN5 viruses detected in China shared genetic similarities and formed several sublineages but were genetically different from the H12N5 virus detected in this study. The NA gene of the SD/W6808 virus very likely originated from the migratory duck H12N5-like viruses detected in Russia (Figure 4).

**Figure 5:**
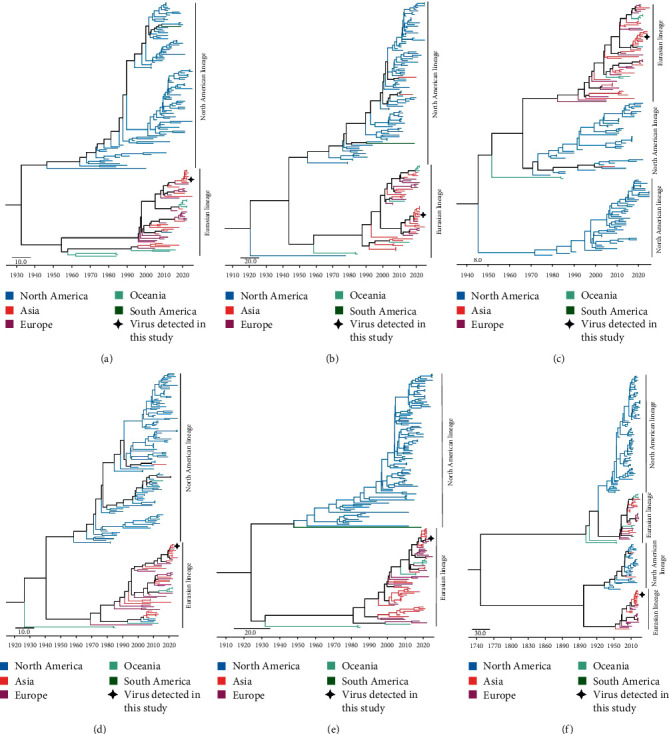
Phylogenetic trees of the internal genes of the H12 viruses. (a) PB2, (b) PB1, (c) PA, (d) NP, (e) M, and (f) NS. The viruses detected in North America, Asia, Europe, Oceania, and South America are shown in different colors. The virus detected in this study is specifically noted in each gene segment tree.

We then constructed phylogenetic trees of the six internal genes (PB2, PB1, PA, NP, M, and NS) of H12 viruses to reveal their evolutionary trends and the reassortment events of the SD/W6808 virus. The phylogenetic analysis indicated that the internal genes of the H12 viruses formed the Eurasian lineage and North American lineage. The PB2, PB1, NP, and M genes clustered into two distinct lineages. The viruses detected in North America and South America mainly clustered into the North American lineage, while the viruses detected in Europe, Asia, and Oceania primarily formed the Eurasian lineage (Figures 5(a), 5(b), 5(d), and 5(e)). The PA genes of the H12 viruses detected in Europe, Asia, and Oceania formed one lineage, while the North American H12 viruses clustered into two distinct sublineages (Figure 5(c)). The NS genes of the H12 viruses circulating in Europe, Asia, and Oceania have evolved into two different sublineages, and the North American viruses also formed two sublineages (Figure 5(f)). Notably, some Eurasian viruses were clustered into the North American lineage, and some North American viruses were placed within the Eurasian lineage in the trees, suggesting that H12 viruses can be transmitted globally by their migratory reservoirs (Figure 5(a)–5(f)). The six internal genes of the H12N5 virus, SD/W6808, detected in this study clustered with the Eurasian lineage. Importantly, the internal genes of the SD/W6808 virus share high nucleotide identity with those of the wild bird H3N8, H6N2, H7N4, H7N7, H9N2, H10N8, H11N2, H12N2, and H12N5 viruses or the duck and human H5N6 viruses detected in Eastern China, suggesting that the SD/W6808-like internal gene segments can flow with migratory birds and have a potential risk of spillover into humans (Figure 5 and Figure S1). Notably, wild bird H3N8, H9N2, and H10N8 viruses, which shared high genetic similarity with SD/W6808, were detected in the YRD wetland between 2019 and 2020 (Figure S1) [9, 31, 32]. These genetic analyses implied that the H12N5 viruses have undergone complicated reassortment and that the circulated viruses detected on the EAA flyway, including the viruses detected in the YRD wetland, contributed to the emergence of the SD/W6808 H12N5 virus.

### 3.4. Identification and Isolation of an H12N5 Virus in Wild Ducks in Eastern China

The YRD wetlands are located in the Shandong Province of Eastern China and are the largest nascent wetland ecosystem in China, providing an ideal habitat and transit area for millions of migratory birds on the EAA Flyway and Western Pacific Flyway. We conducted annual AIV surveillance in the YRD wetland and collected 1,151 fresh fecal droppings from migratory wild ducks on October 28, 2019. An H12N5 virus, A/wild duck/Shandong/W6808/2019 (H12N5) (SD/W6808) (Table 1) (Accession Number in GISAID: EPI2756323-EPI2756327, EPI2756329-EPI2756331), plus one H1N1 virus, and three H4N6 viruses were isolated from the samples and identified.

### 3.5. Molecular Characteristics of SD/W6808

We analyzed the molecular characteristics of the H12N5 virus used in this study, and the motif PQVQNR.GLF with no multiple consecutive basic amino acid insertion was observed at the cleavage site in HA of SD/W6808, which is typical for low pathogenicity in chickens. Amino acid substitutions that conferred increased binding specificity to the human-type receptor were not observed in the HA of SD/W6808. The amino acid mutations in six internal genes that contribute to enhanced replication and virulence in mammals were also analyzed. Classical mutations, such as PB2-E627K or PB2-D701N [38, 39], were not found; however, N30D and T215A in M1 and I106M in NS1 [40–42], which contribute to the enhanced pathogenicity of AIVs in mammals, were found in SD/W6808. Consequently, the H12N5 virus isolated from wild ducks has not acquired mammal-adapted mutations (Table S1).

### 3.6. Infectivity and Replication of SD/W6808 in Ducks and Chickens

**Figure 6:**
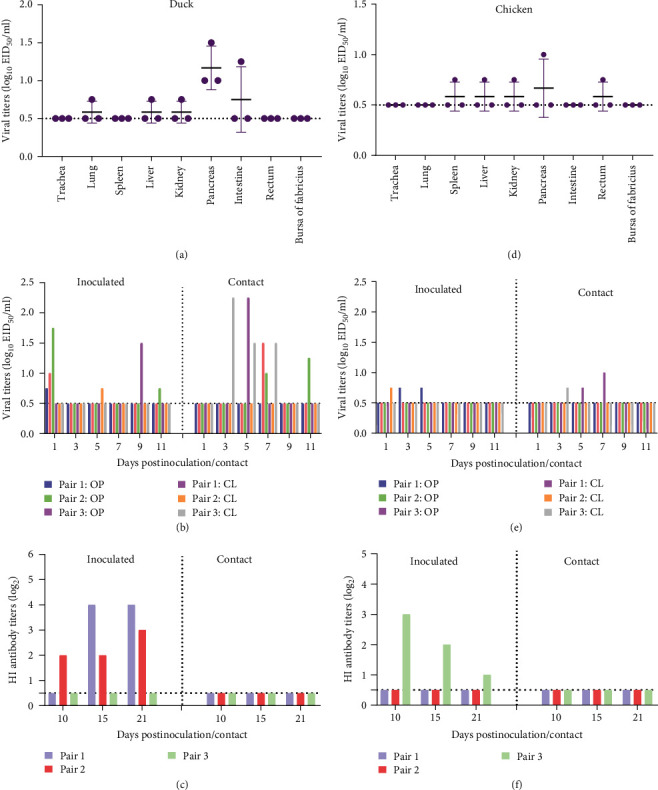
Infectivity and replication of the SD/W6808 virus in ducks and chickens. (a) Replication of SD/W6808 in ducks. (b) Transmission of SD/W6808 in ducks. (c) HI antibody in the serum of the ducks. (d) Replication of SD/W6808 in chickens. (e) Transmission of SD/W6808 in chickens. (f) HI antibody in the serum of the chickens. The dashed lines in each panel indicate the lower detection limit.

According to the summarized and categorized data in this study, the birds of *Anatidae* and *Scolopacidae* are the central reservoirs of H12N5 viruses. We first evaluated whether this wild duck-originating H12N5 virus can infect SPF ducks. The SD/W6808 virus was detected in the pancreas of the three inoculated ducks, with a viral titer of 1–1.5 log_10_ EID_50_/ml. However, only one of the three birds was positive for the virus in the lung, liver, kidney, and intestine, with viral titers ranging from 0.75 to 1.25 log_10_ EID_50_/ml. No virus was detected in the trachea, spleen, rectum, or bursa of Fabricius (Figure 6(a)). Viral titers of oropharyngeal or cloacal swabs of the inoculated and contact ducks were measured. The viruses were detected in all the birds of both the inoculated and contact groups, with viral titers ranging from 0.75 to 2.25 log_10_ EID_50_/ml (Figure 6(b)). The infectivity studies indicated that the H12N5 virus has the potential to infect domestic ducks, although the replication of the virus in ducks was not good.

To date, chickens have not been reported to be infected with H12 viruses. We further tested whether this wild duck-originating H12N5 virus can infect chickens. The virus was detected in the spleen, liver, kidney, pancreas, and rectum of one of the three inoculated chickens, with viral titers of 0.75–1.0 log_10_ EID_50_/ml. No virus was detected in the trachea, lung, intestine, or bursa of Fabricius (Figure 6(d)). Limited virus shedding was detected in oropharyngeal or cloacal swabs of the inoculated or contact chickens, indicating the low transmission efficiency of the H12N5 virus in chickens (Figure 6(e)). These results indicate a fecal-oral route may be more effective for the H12N5 viruses in birds. Notably, HI antibody was not detected in any of the contact ducks or chickens, although virus shedding was positive in two or three birds of the contact groups (Figures 6(c) and 6(f)). These experimental infection studies implied that the wild duck-originating H12N5 virus has not adapted to replicate efficiently in both ducks and chickens.

### 3.7. Replication of SD/W6808 in Mice

**Figure 7:**
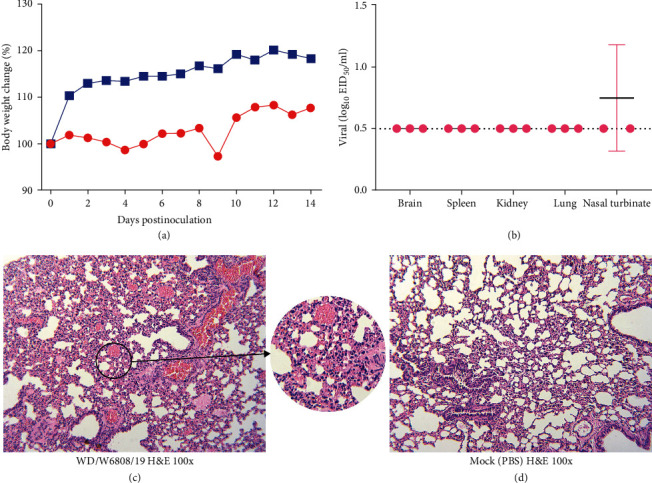
Replication of SD/W6808 virus in mice. Mice were euthanized on day 3 p.i. with 10^6^ EID_50_ of the test virus, and the organs, including the lungs, were sampled for viral titration and pathological studies (H&E staining). The images of the pathological staining were taken at ×100 magnification. (a) Body weight change of the mice. (b) Replication of SD/W6808 virus in mice. The dashed line indicates the lower detection limit. (c) Pathological lesions (H&E staining) of the lung caused by SD/W6808. The arrow mark indicates the lesions caused by the inoculated virus. (d) H&E staining of the lungs of mice inoculated with PBS.

To uncover the mammalian adaptation of this wild bird-originating virus, mice were used to test the replication and virulence of the H12N5 virus. The WD/W6808 virus exhibited very limited replication ability in mice, with 1.25 log_10_ EID_50_/ml in the nasal turbinate of one of three inoculated mice. No virus was detected in the lung, spleen, kidney, or brain (Figure 7(b)). The mice lost 2.72% of their body weight during the observation period (Figure 7(a)). Pathological studies showed that the WD/W6808 virus contributed to mild lesions in the lung, including the widening of the alveolar septa and narrowing of the alveolar lumen (Figures 7(c) and 7(d)). These results implied that prior adaptation was essential for the wild duck-originating H12N5 virus to achieve good replication in mice.

## 4. Discussion

As one of the 16 HA subtypes of AIVs, H12 viruses are mainly found in wild birds and have received little attention due to their low pathogenicity in these animals. Since the first detection in wild birds in 1975, only 501 strains of H12 viruses have been reported or submitted to databases, which is significantly lower than the number of common subtypes. Previous 10 years active monitoring of waterfowl in Russia also found the H12 virus was not frequently isolated compared with the total AIV isolation rate [21]. The HA gene of the H12 viruses seems to have a combination bias with the N5 gene because 58.68% of the identified H12 strains are H12N5 viruses. Although H12N5 viruses have been detected on five continents, the North American viruses make up the majority of the identified strains [43–45]. Several determinants, including the surveillance network, bird species and number, and established migratory flyway, may collectively contribute to the detection of H12 viruses in birds [46]. The H12N5 viruses were mainly detected in birds of *Anatidae* and *Scolopacidae*, although they have been found in different waterfowl and shorebirds. The diversity of the reservoirs of the H12N5 viruses was similar to that of avian H3N8 viruses but was different from that of H16 viruses, which are rare subtypes of AIVs [31, 47, 48]. In our previous study, the summarized and categorized data indicated that ducks are the central reservoirs of H3N8 AIVs, while migratory gulls are the primary reservoirs of H16 viruses [31, 32].

The temporal and spatial distributions of viruses are closely related to bird populations and their migratory flyways [49, 50]. The H12 viruses isolated from America are genetically different from the viruses detected from Europe, Asia, and Oceania, although reassortment was observed between the North American lineage and the Eurasian lineage [21]. For instance, the HA gene of the H12N5 virus in this study shared a high identity with the H12N8 virus detected in Shanghai of Eastern China by Tang et al. [22]. As an important wetland in Eastern China, the YRD wetland is an ideal habitat for birds on the EAA flyway. The viruses isolated from different bird populations during a surveillance period will contribute to our understanding of the evolution of the viruses in their reservoirs. The internal genes of this H12N5 virus shared a high genetic similarity with those of the wild bird-originated H3N8, H9N2, and H10N8 viruses detected in the YRD wetland. Genetic identities similar to those of the viruses detected in the YRD wetland were also found in the wild bird H3N8 viruses in our previous studies [32]. Reassortment or gene shifting between different subtypes in their reservoirs may play a significant role in the maintenance of viruses in the gene pool of AIVs, especially the maintenance of rare subtypes [51]. Long-term surveillance, sufficient sample numbers from representative birds, and successful virus identification in the YRD wetland will collectively contribute to advance our understanding of the evolution of AIVs in migratory birds.

The spillover of AIVs into poultry and mammals from their natural reservoirs is a prerequisite for potential endemics [34, 52]. The infectivity of the H12 viruses, belonging to a rare subtype, to domestic birds has been little evaluated and is thus largely unknown. In a previous study, a wild mallard-originating H12 virus was reported to replicate poorly in experimental mallards [53]. The wild bird-origin H12N2 and H12N5 viruses isolated in Russia were tested in chickens and exhibited low pathogenic for chickens [21]. In this study, we used SPF ducks to test the replication, viral shedding, and transmission of the wild duck-originating H12N5 virus. The negative or low positive viral titers of the H12N5 virus in the collected tissue or organs implied that the H12N5 virus used in this study has poor adaptation to domestic ducks. Despite the limited replication in ducks, viral shedding and transmission of the virus between the inoculated and contact birds could still be detected. Previously, there were few reports on the infectivity and replication of H12 viruses in chickens, and H12 viruses have not been identified in chickens. The infectivity experiments in this study indicated that SPF chickens are not susceptible to this wild duck-originating H12 virus. Additionally, the H12 virus exhibited poor viral shedding and transmissibility in chickens, in contrast to the results observed for the viruses in ducks. We previously found that wild bird-originating H10 viruses exhibited good adaptation to both ducks and chickens, while H3N8 viruses isolated from wild birds showed good replication in ducks but poor adaptation in chickens [31, 32]. However, the rare subtypes of AIVs, such as the H12 virus in this study and H16 in our previous report, replicated poorly in both ducks and chickens. Additionally, the H12N5 virus has not adapted to replicate in mice and exhibited low pathogenicity in mice, similar to the findings of the H12N2, H12N5, and H16N3 virus in mice [21, 31]. Consequently, further studies are essential to understand the molecular basis of host adaptation in wild bird-originating influenza viruses.

In conclusion, we performed a detailed analysis to understand the ecology of H12 viruses and mapped the evolutionary dynamics of H12N5 viruses. Animal studies indicated that the H12N5 virus isolated from wild ducks has poor adaptation to domestic birds and mammals. These results emphasize that strengthened annual surveillance along migratory flyways is essential for monitoring rare subtypes of AIVs.

## Figures and Tables

**Table 1:** The information of all the deposited H12 viruses in China.

No.	Isolate name	Subtype	Location	Host	Isolate ID/accession	Collection date
1	A/wild goose/Dongting/C1037/2011	H12N8	Hunan	Wild goose	EPI_ISL_140706	2011-11-10
2	A/environment/Hunan/S4484/2011	H12N7	Hunan	Environment	EPI_ISL_143956	2011-11-13
3	A/*Mareca falcata*/China/D29/2015	H12N2	China	*M. falcata*	MK301256.1–MK301263.1	2015-02-06
4	A/Environment/Jiangxi/08488/2015	H12N2	Jiangxi	Environment	EPI_ISL_247287	2015-11-19
5	A/common teal/Shanghai/NH101807/2017	H12N2	Shanghai	Common teal	EPI_ISL_499092	2017-10-18
6	A/mallard/Shanghai/JDS110851/2017	H12N5	Shanghai	Mallard	EPI_ISL_499093	2017-11-08
7	A/mallard/Shanghai/NH011204/2018	H12N5	Shanghai	Mallard	EPI_ISL_499094	2018-01-12
8	A/common teal/Shanghai/NH102615/2018	H12N2	Shanghai	Common teal	EPI_ISL_499096	2018-10-26
9	A/common teal/Shanghai/NH110165/2018	H12N2	Shanghai	Common teal	EPI_ISL_499097	2018-11-01
10	A/common teal/Shanghai/NH112319/2018	H12N2	Shanghai	Common teal	EPI_ISL_499095	2018-11-23
11^a^	A/wild duck/Shandong/W6808/2019	H12N5	Shandong	Wild duck	EPI_ISL_18300245	2019-10-28
12	A/common teal/Shanghai/JDS110203/2019	H12N8	Shanghai	Common teal	EPI_ISL_501427	2019-11-02

The sequences of these H12 viruses are available from GISAID or Genbank. ^a^The H12N5 virus detected in this study.

## Data Availability

The sequences data used to support the findings of this study are included within the article and have been deposited in GISAID (EPI2756323–EPI2756327 and EPI2756329–EPI2756331).

## Supplementary Materials

Supplementary Materials Figure S1: phylogenetic trees of PB2 (A), PB1 (B), PA (C), NP (D), M (E), and NS (F) genes of H12N5 viruses. Table S1: molecular characteristics of the H12N5 virus in this study.
