# Supplementary material for: Ecological and Genetic Landscapes of Global H12 Avian Influenza Viruses and Biological Characteristics of an H12N5 Virus Isolated from Wild Ducks in Eastern China
Source: Transbound Emerg Dis. 2024 Feb 22;2024:9140418. doi: 10.1155/2024/9140418 (PMC12017136; doi:10.1155/2024/9140418)
Supplement: Supplementary Materials — Figure S1: phylogenetic trees of PB2 (A), PB1 (B), PA (C), NP (D), M (E), and NS (F) genes of H12N5 viruses. Table S1: molecular characteristics of the H12N5 virus in this study. [file 9140418.f1.pdf]

Figure S1 A PB2

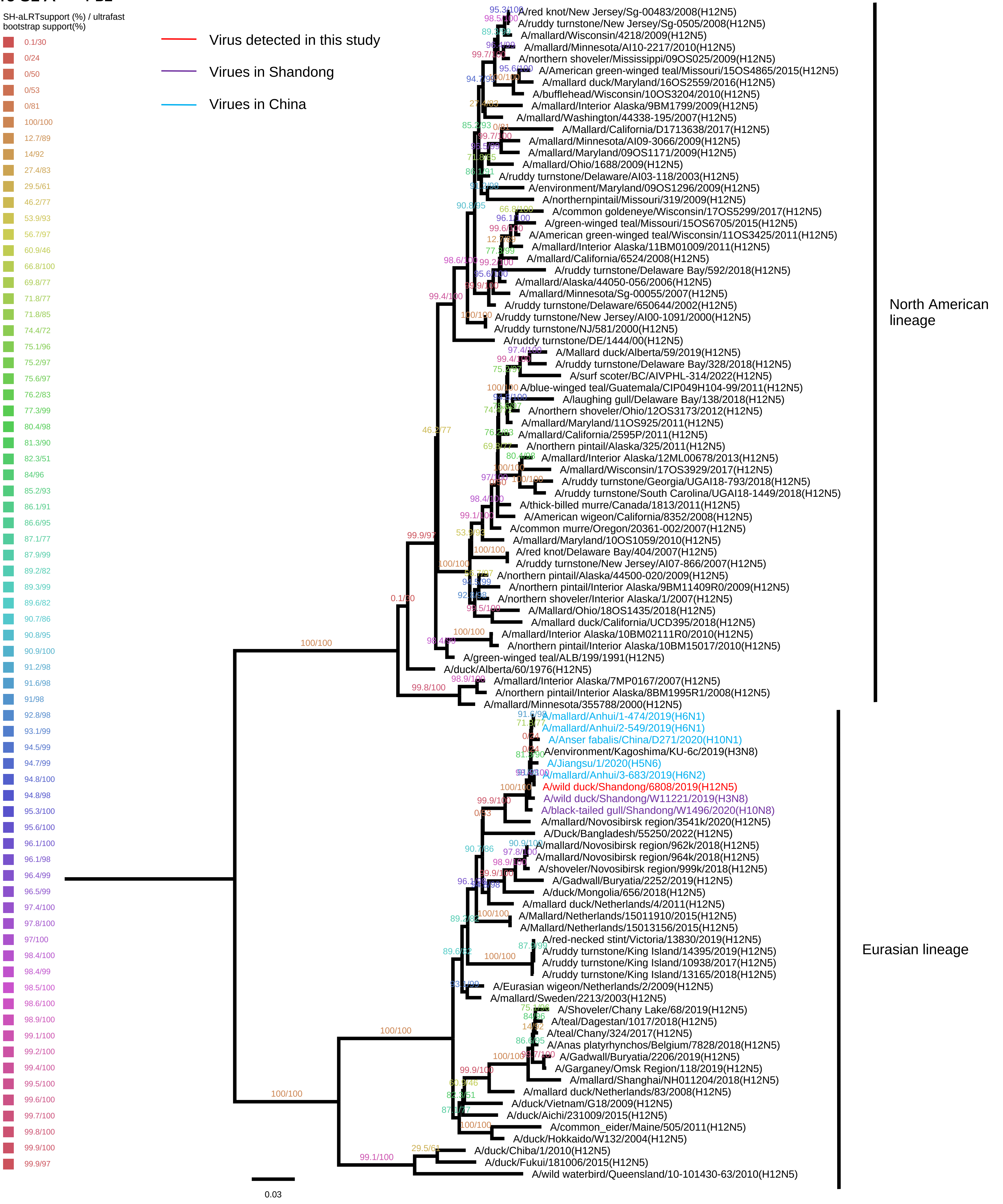

Figure S1 B PB1

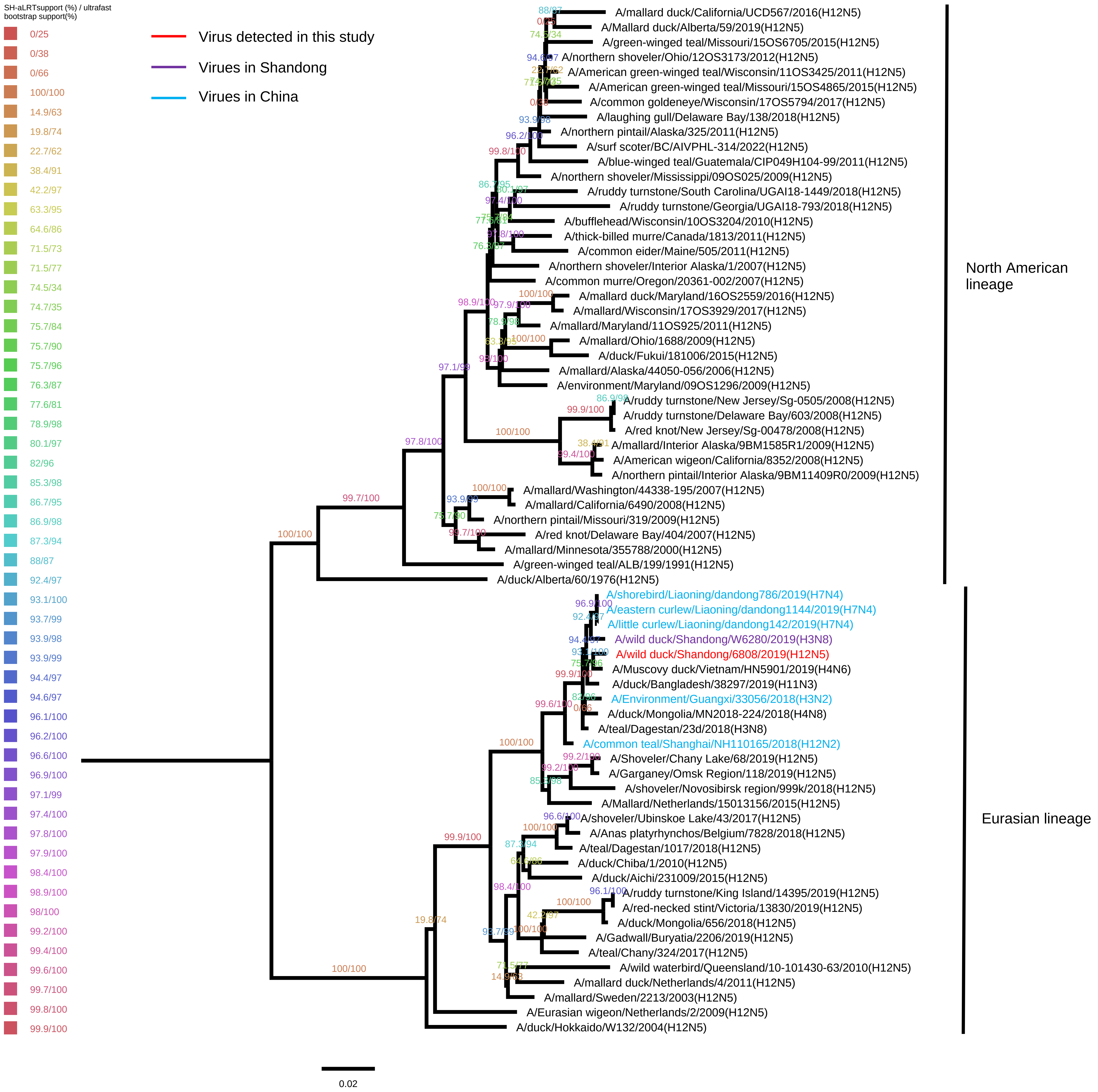

Figure S1 C PA

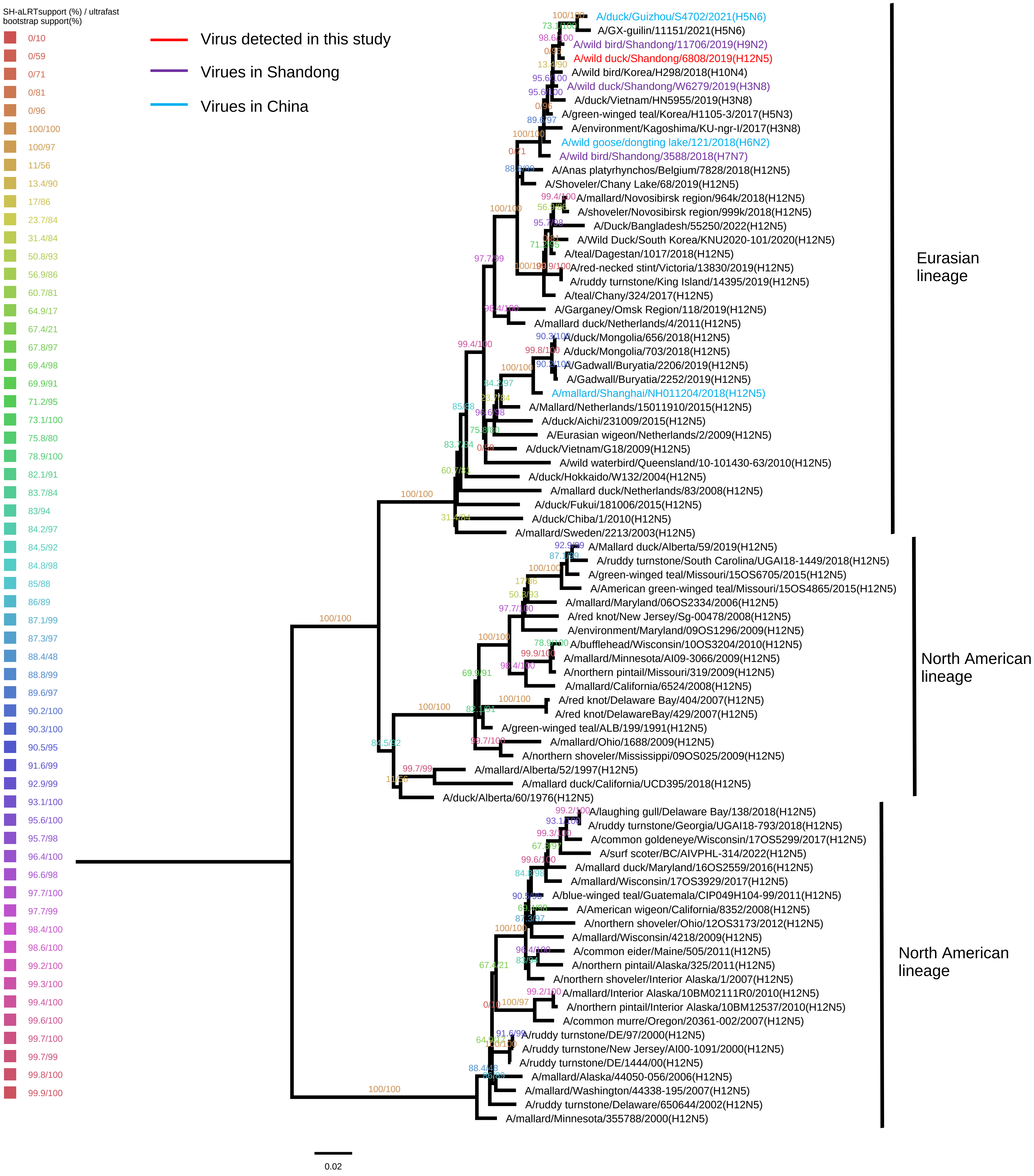

Figure S1 D NP

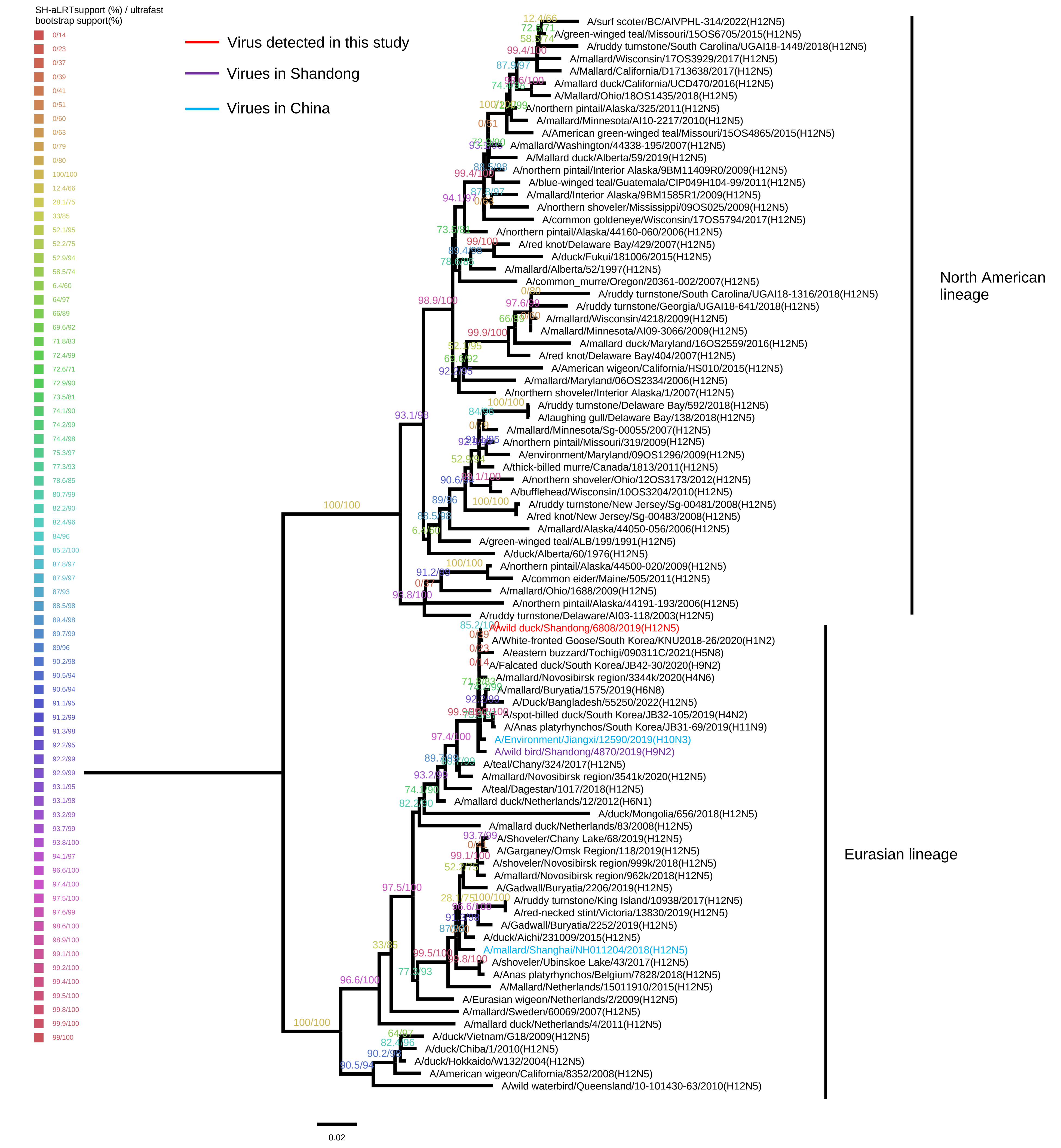

Figure S1 E M

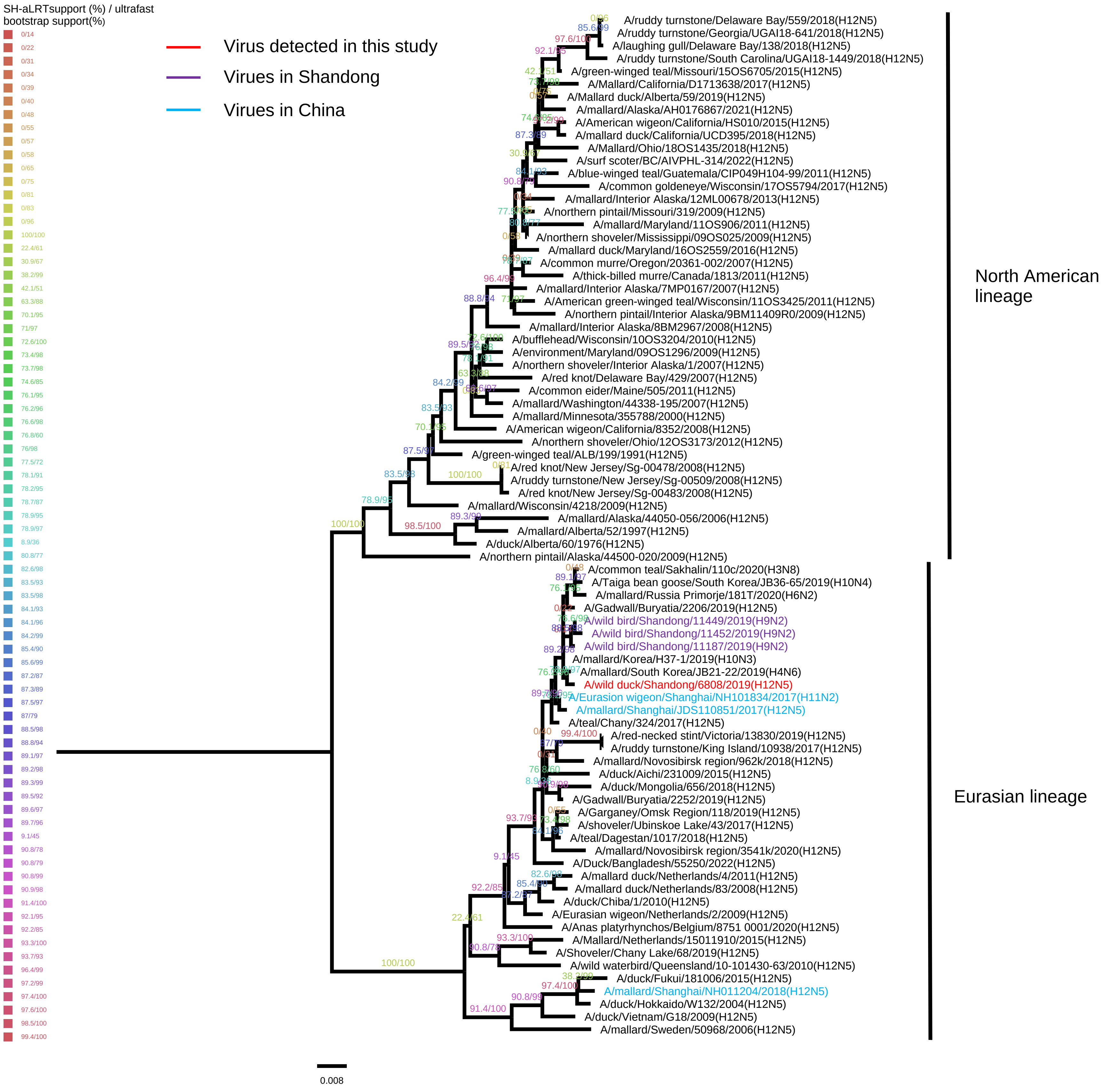
SH-aLRTsupport (%) / ultrafast bootstrap support(%)
0/14
0/22
0/31
0/34
0/39
0/40
0/48
0/55
0/57
0/58
0/65
0/75
0/81
0/83
0/96
100/100
22.4/61
30.9/67
38.2/99
42.1/51
63.3/88
70.1/95
71/97
72.6/100
73.4/98
73.7/98
74.6/85
76.1/95
76.2/96
76.6/98
76.8/60
76/98
77.5/72
78.1/91
78.2/95
78.7/87
78.9/95
78.9/97
8.9/36
80.8/77
82.6/98
83.5/93
83.5/98
84.1/93
84.1/96
84.2/99
85.4/90
85.6/99
87.2/87
87.3/89
87.5/97
87/79
88.5/98
88.8/94
89.1/97
89.2/98
89.3/99
89.5/92
89.6/97
89.7/96
9.1/45
90.8/78
90.8/79
90.8/99
90.9/98
91.4/100
92.1/95
92.2/85
93.3/100
93.7/93
96.4/99
97.2/99
97.4/100
97.6/100
98.5/100
99.4/100
Virus detected in this study
Virues in Shandong
Virues in China
A/ruddy turnstone/Delaware Bay/559/2018(H12N5)
A/ruddy turnstone/Georgia/UGAI18-641/2018(H12N5)
A/laughing gull/Delaware Bay/138/2018(H12N5)
A/ruddy turnstone/South Carolina/UGAI18-1449/2018(H12N5)
A/green-winged teal/Missouri/15OS6705/2015(H12N5)
A/Mallard/California/D1713638/2017(H12N5)
A/Mallard duck/Alberta/59/2019(H12N5)
A/mallard/Alaska/AH0176867/2021(H12N5)
A/American wigeon/California/HS010/2015(H12N5)
A/mallard duck/California/UCD395/2018(H12N5)
A/Mallard/Ohio/18OS1435/2018(H12N5)
A/surf scoter/BC/AIVPHL-314/2022(H12N5)
A/blue-winged teal/Guatemala/CIP049H104-99/2011(H12N5)
A/common goldeneye/Wisconsin/17OS5794/2017(H12N5)
A/mallard/Interior Alaska/12ML00678/2013(H12N5)
A/northern pintail/Missouri/319/2009(H12N5)
A/mallard/Maryland/11OS906/2011(H12N5)
A/northern shoveler/Mississippi/09OS025/2009(H12N5)
A/mallard duck/Maryland/16OS2559/2016(H12N5)
A/common murre/Oregon/20361-002/2007(H12N5)
A/thick-billed murre/Canada/1813/2011(H12N5)
A/mallard/Interior Alaska/7MP0167/2007(H12N5)
A/American green-winged teal/Wisconsin/11OS3425/2011(H12N5)
A/northern pintail/Interior Alaska/9BM11409R0/2009(H12N5)
A/mallard/Interior Alaska/8BM2967/2008(H12N5)
A/bufflehead/Wisconsin/10OS3204/2010(H12N5)
A/environment/Maryland/09OS1296/2009(H12N5)
A/northern shoveler/Interior Alaska/1/2007(H12N5)
A/red knot/Delaware Bay/429/2007(H12N5)
A/common eider/Maine/505/2011(H12N5)
A/mallard/Washington/44338-195/2007(H12N5)
A/mallard/Minnesota/355788/2000(H12N5)
A/American wigeon/California/8352/2008(H12N5)
A/northern shoveler/Ohio/12OS3173/2012(H12N5)
A/green-winged teal/ALB/199/1991(H12N5)
A/red knot/New Jersey/Sg-00478/2008(H12N5)
A/ruddy turnstone/New Jersey/Sg-00509/2008(H12N5)
A/red knot/New Jersey/Sg-00483/2008(H12N5)
A/mallard/Wisconsin/4218/2009(H12N5)
A/mallard/Alaska/44050-056/2006(H12N5)
A/mallard/Alberta/52/1997(H12N5)
A/duck/Alberta/60/1976(H12N5)
A/northern pintail/Alaska/44500-020/2009(H12N5)
North American lineage
A/common teal/Sakhalin/110c/2020(H3N8)
A/Taiga bean goose/South Korea/JB36-65/2019(H10N4)
A/mallard/Russia Primorje/181T/2020(H6N2)
A/Gadwall/Buryatia/2206/2019(H12N5)
A/wild bird/Shandong/11449/2019(H9N2)
A/wild bird/Shandong/11452/2019(H9N2)
A/wild bird/Shandong/11187/2019(H9N2)
A/mallard/Korea/H37-1/2019(H10N3)
A/mallard/South Korea/JB21-22/2019(H4N6)
A/wild duck/Shandong/6808/2019(H12N5)
A/Eurasion wigeon/Shanghai/NH101834/2017(H11N2)
A/mallard/Shanghai/JDS110851/2017(H12N5)
A/teal/Chany/324/2017(H12N5)
A/red-necked stint/Victoria/13830/2019(H12N5)
A/ruddy turnstone/King Island/10938/2017(H12N5)
A/mallard/Novosibirsk region/962k/2018(H12N5)
A/duck/Aichi/231009/2015(H12N5)
A/duck/Mongolia/656/2018(H12N5)
A/Gadwall/Buryatia/2252/2019(H12N5)
A/Garganey/Omsk Region/118/2019(H12N5)
A/shoveler/Ubinskoe Lake/43/2017(H12N5)
A/teal/Dagestan/1017/2018(H12N5)
A/mallard/Novosibirsk region/3541k/2020(H12N5)
A/Duck/Bangladesh/55250/2022(H12N5)
A/mallard duck/Netherlands/4/2011(H12N5)
A/mallard duck/Netherlands/83/2008(H12N5)
A/duck/Chiba/1/2010(H12N5)
A/Eurasian wigeon/Netherlands/2/2009(H12N5)
A/Anas platyrhynchos/Belgium/8751 0001/2020(H12N5)
A/Mallard/Netherlands/15011910/2015(H12N5)
A/Shoveler/Chany Lake/68/2019(H12N5)
A/wild waterbird/Queensland/10-101430-63/2010(H12N5)
A/duck/Fukui/181006/2015(H12N5)
A/mallard/Shanghai/NH011204/2018(H12N5)
A/duck/Hokkaido/W132/2004(H12N5)
A/duck/Vietnam/G18/2009(H12N5)
A/mallard/Sweden/50968/2006(H12N5)
Eurasian lineage
0.008

Figure S1 F NS

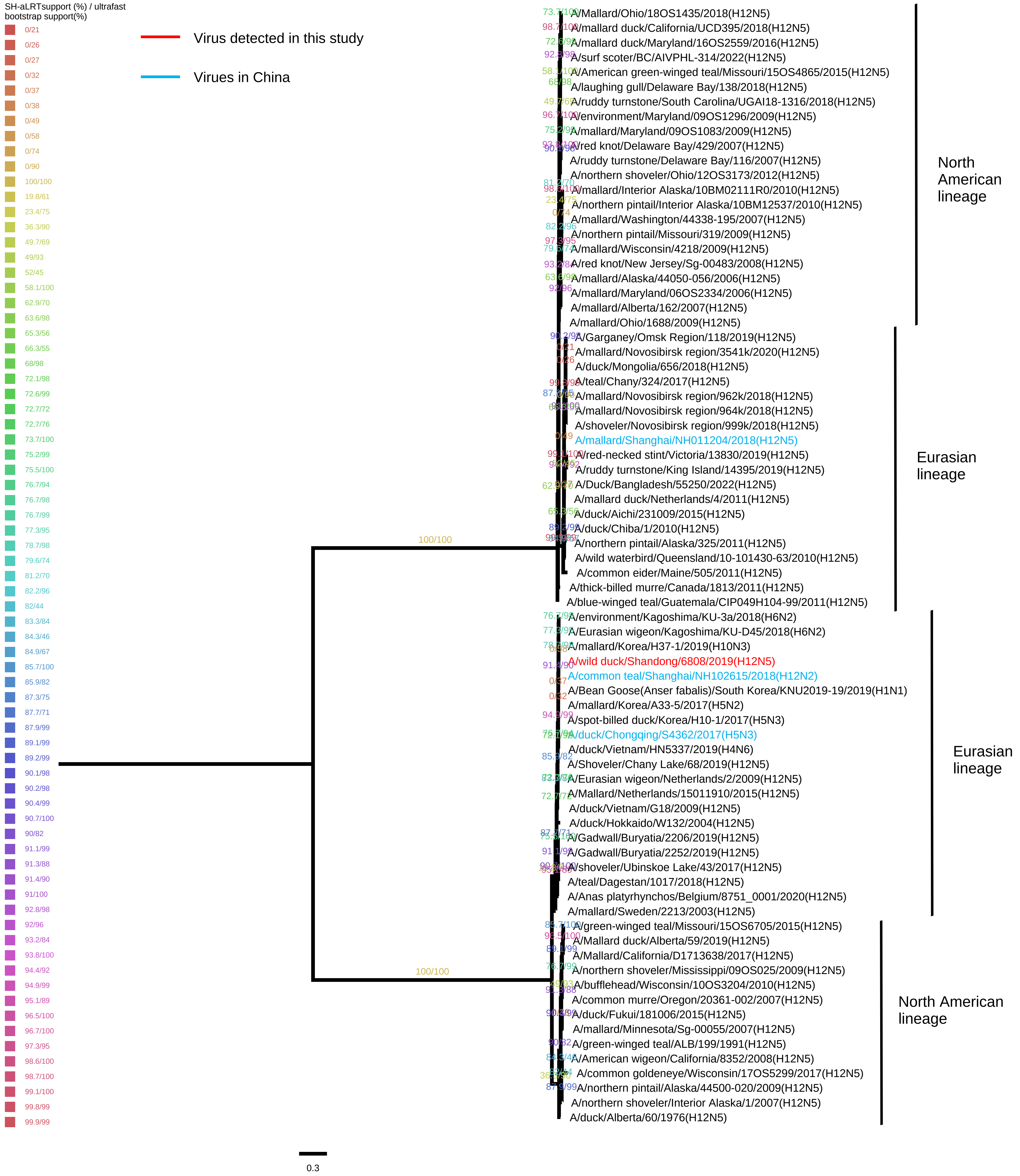

Figure S1. Phylogenetic trees of PB2 (A), PB1 (B), PA (C), NP (D), M (E), and NS (F) genes of H12N5 viruses. The sequence name colored in red was the H12N5 virus detected in this study, the sequences name in purple were the virues in Shandong,and the sequences name in blue were the virues in China.

Table S1. Molecular characteristics of the H12N5 virus in this study

Protein	Mutation	Virus
		WD/W6808/19
HA	Cleavage site	^{337}PQVQNR.GLF^{345}
	Q226L[a]	Q
	G228S	G
PB2	E627K	E
	D701N	D
	A453S	A
PB1	K198I	K
	Y436H	Y
PA	L13P	P
	V63I	V
M1	N30D	D
	T215A	A
M2	S31N	S
NS1	D92E	D
	P42S	A
	I106M	M

[a] H3 numbering
